# Experimental and Modeling Study of the Evolution of Mechanical Properties of PAN-Based Carbon Fibers at Elevated Temperatures

**DOI:** 10.3390/ma12050724

**Published:** 2019-03-01

**Authors:** Chenggao Li, Guijun Xian

**Affiliations:** 1 Key Lab of Structures Dynamic Behavior and Control (Harbin Institute of Technology), Ministry of Education, Harbin 150090, China; lichenggao02@126.com; 2 Key Lab of Smart Prevention and Mitigation of Civil Engineering Disasters of the Ministry of Industry and Information Technology, Harbin Institute of Technology, Harbin 150090, China; 3 School of Civil Engineering, Harbin Institute of Technology, Harbin 150090, China

**Keywords:** carbon fiber, elevated temperature, mechanical properties, rule of mixtures, elastic mechanics theory

## Abstract

In the present article, the degradation of the tensile properties of polyacrylonitrile (PAN)-based carbon fibers at elevated temperatures in air was studied experimentally and modeled. The tensile properties, mass loss, surface morphology, and elements and functional groups of carbon fibers were characterized. It can be concluded that the tensile strength and modulus of the carbon fibers decreased remarkably when the exposure temperature exceeded 500 °C. Oxidation at elevated temperatures etched the carbon layer from the skin to the core of the carbon fibers, leading to mass loss. According to the rule of mixtures, an exponential decay model was put forward to describe the degradation behavior of tensile modulus exposed to different temperatures and times. The thickness of the outer layer (*T_outer_*) of carbon fibers was obtained to be 0.818 μm. The ultimate exposure temperature was predicted to be 699.4 °C for 30 min, and the ultimate exposure time was 13.2 h at 500 °C. Furthermore, the time–temperature equivalence equation of tensile modulus was deduced. Through the introduction of the normalized oxidation degree, a degradation model of the tensile modulus at any exposure temperature (~800 °C) and time (~800 min) was also proposed. From the elastic mechanics theory for anisotropic solids, the degradation model of tensile strength exposed to elevated temperature was confirmed. It can be observed that the proposed model had good agreement with the experimental results.

## 1. Introduction

Carbon fiber-reinforced polymer (CFRP) composites are attractive for load-bearing structures in many engineering fields due to their superior mechanical properties and potentially high durability [1,2]. In recent years, the application of CFRP in different shapes (e.g., sheets, grids, and tendons) for strengthening or reinforcing civil engineering structures has increased [3,4]. Among these applications, the fire-resistance performance of CFRPs is a major challenge [5,6,7,8], which mainly stems from the low temperature and poor flame resistances of the polymer matrices [9,10] and may even be attributable to the degradation of carbon fibers at elevated temperatures (e.g., oxidation or decomposition). Furthermore, existing studies have shown that the degradation of carbon fibers may have certain priorities compared to the decomposition of polymer matrices [11].

The oxidation of carbon fiber at elevated temperatures in air is a key factor inducing the degradation of the material properties (e.g., thermal stability and tensile properties) [12,13,14]. Furthermore, the degradation is generally accepted to be as a result of changes in the microstructures of carbon fibers during oxidation [15,16]. Some research works have focused on the degradation of carbon fiber for heat-resistant carbon–carbon composites at high temperatures (>1400 °C) [17,18]. Liu et al. [19] qualitatively studied the effect of the microstructure of PAN-based carbon fibers on mechanical properties during high-temperature graphitization (1800–2800 °C). The results showed the crystallite size, interlayer spacing, and crystallite preferred orientation degree changed dramatically with increasing temperature. Meanwhile, the degree of skin–core structure in the carbon fibers further intensified. Finally, it was concluded that the tensile strength had and indirect association with the crystallite size and degree of skin–core structure, while the tensile modulus depended on the crystallite size and surface ordering. Wang et al. [20] found that heat treatment at 1400 °C decreased the surface activity of the carbon fiber by reducing the oxygen and nitrogen atoms and increasing the carbon fraction. The graphitization degree can be improved by heat treatment, by decreasing the interlayer spacing and increasing crystallite dimensions. The tensile strength remained unchanged after heat treatment, owing to the limited changes in the microstructure caused by heat treatment. 

When CFRP composite suffers from an actual fire [21,22], the carbon fibers on the surfaces of the CFRP close to the fire are considered to be exposed to an oxygen-rich environment. The heat generated from a fire will transfer to the CFRP surface far away from the fire via radiation or convection [23]. The temperature range of 400–1100 °C from radiation or convection is considered as the practical exposure temperature in fire conditions [24]. So far, few studies have focused on the degradation law and mechanism in this temperature range. Feih et al. [25] found that the mass loss of carbon fiber in air initially decreased when the temperature exceeded 500–550 °C, owing to oxidation. Meanwhile, the Young’s modulus of the carbon fiber decreased by about 20% when the temperatures increased from 400 to 700 °C, while the tensile strength reduced by nearly 40%. They concluded that the degradation of tensile properties was attributed to the skin–core heterogeneity of the microstructure for carbon fiber. Considering the rule of mixtures, the outer layer thickness *T_outer_* and Young’s modulus of carbon fiber were estimated to be around 0.65 μm and 315 GPa, respectively. Yin et al. [26] obtained a linear relationship between the carbon fiber diameter and the exposure time when exposure temperature changed from 550 to 860 °C. They found that a white-colored residue appearing after full oxidation had affected the reaction rate once the oxidation reached the central part of the fiber, which suggested the crystal structure or compositions of the fiber cores were different from the rest of the fibers. In summary, the study of the elevated temperature properties of carbon fiber is essential to simulate the actual exposure of carbon fibers to a fire. Furthermore, an approximate degradation model of carbon fiber in air is significant to predict the residual properties of CFRP after fire.

Based on the recent studies, the exposure conditions of CFRPs at elevated temperatures for engineering applications are summarized in Table 1 to simulate the actual fire condition, including the composite type, exposed temperature range and time, the fire protection coating, etc. As shown in Table 1, when CFRPs were applied in engineering structures to strengthen or repair concrete components, the potential resistance to elevated temperature was larger owing to the fire protection coating. Meanwhile, this also provided a reference for the selection of the exposure temperature and time of carbon fibers at elevated temperatures in this paper.

In the present work, the degradation of tensile properties for carbon fibers exposed to elevated temperature (~700 °C) and time (~10 h) was studied and modeled. The mass loss, surface morphology, and elements and functional groups of carbon fibers were tested. Furthermore, the degradation mechanism of the carbon fibers was revealed. The rule of mixtures was applied to deduce the degradation of tensile modulus. It was predicted that the ultimate exposure temperature was 699.4 °C for 30 min and the ultimate exposure time was 13.2 h at 500 °C. Furthermore, the time–temperature equivalence equation was put forward to analyze the degradation behavior of tensile modulus. Combining the elastic mechanics theory for anisotropic solids, the degradation model of tensile strength exposed to elevated temperature was confirmed. The agreement between theory and experiment was verified through the carbon fiber tensile results.

## 2. Experimental

### 2.1. Raw Materials

The PAN-based carbon fibers were produced by the Plastics Group (TC36S, Taiwan). The nominal tensile strength and modulus of carbon fiber were 4.7 and 240 GPa, respectively. 

### 2.2. Elevated Temperature Exposures

Carbon fibers were cut with lengths of about 10 cm and then exposed to temperatures of 400–700 °C for 30 min, or 1–10 h at 500 °C, or 10 h at 300 °C in a muffle furnace at a heating rate of 10 °C/min under an oxygen-rich environment. By contrast, the control carbon fiber was exposed to air at room temperature. Note that the selected elevated temperatures are considered as actual exposure temperatures when CFRPs suffer from a fire, as shown in Table 1.

### 2.3. Tensile Tests

According to ASTM D 3379-75, tensile tests of single carbon fibers were conducted using a JQ03A single-fiber tensile tester (Zhongchen Digital Technic Apparatus Co., Ltd., Shanghai, China). For each condition, about 50 specimens were repeated and the cross-head displacement rate was 0.00125 mm/min [32]. Before and after the elevated temperature exposure, the tensile modulus and tensile strength were verified by measuring the fiber diameters of ten samples with a scanning electron microscope (SEM).

### 2.4. Thermal Gravimetric Analysis (TGA)

The mass evolution of the carbon fibers at elevated temperatures was characterized by TGA (NETZSCH STA 449C, Selb, Germany) at a heating rate of 10 °C/min in air. The test samples were around 10 mg, and 20 mL/min dry air flow was applied during testing.

### 2.5. Surface Morphology

The surface morphology of the carbon fibers before and after the exposures was analyzed and the diameters were measured by scanning electron microscope (SEM, Quanta-200F, FEI, Hillsboro, OR, USA). The roughness of carbon fiber surfaces was characterized by atomic force microscope (AFM, Bruker Corporation, Hamburg, Germany).

### 2.6. Elements and Functional Groups

#### 2.6.1. X-ray Photoelectron Spectroscopy (XPS)

The XPS spectra of carbon fiber samples were acquired using a K-Alpha photoelectron energy spectrometer (Thermo Fisher Scientific Company, Waltham, MA, USA) equipped with an AlKα X-ray (1486.6 eV) source. The X-ray power was set at 200 W. The pressure in the main vacuum chamber was typically 10^−8^ mbar. The survey scans were collected from the binding energy range of 0–1350 eV. The binding energy scale was calibrated by C1s (284.6 eV). The professional XPSPEAK4.1 software (Kratos analytical company, New York, NY, USA) was used to analyze the testing data.

#### 2.6.2. Fourier Transform Infrared Spectroscopy (FTIR)

FTIR spectra of carbon fiber samples were obtained using a FTIR783 (Perkin-Elmer spectrometer, Waltham, MA, USA). The carbon fibers before and after exposures were cut and ground into a pastille form containing 2 mg mixed with 200 mg of KBr. Each spectrum was obtained by scanning the specimens 64 times in the wave number range of 400–4000 cm^−1^ with a resolution of 4 cm^−1^.

## 3. Theoretical Model

### 3.1. Rule-of-Mixture of the Tensile Modulus for the Skin–Core Heterogeneity of Carbon Fiber

**Figure 1:**
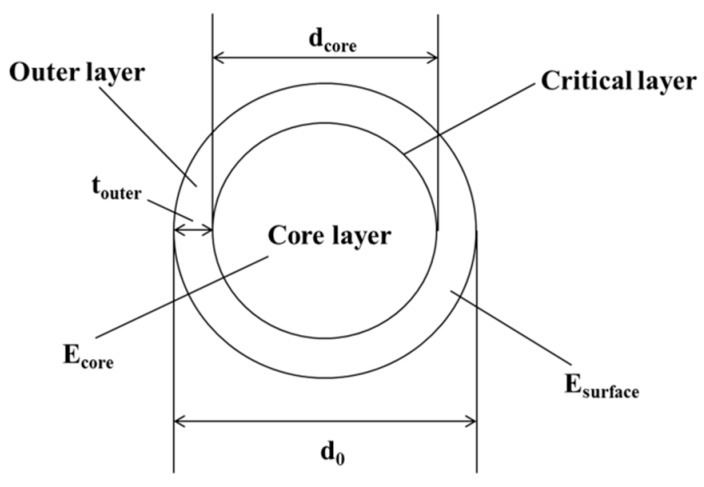
Skin–core structure model of carbon fiber.

The uniform surface oxidation at elevated temperature decreased the diameter of fiber and revealed the skin–core feature of carbon fibers, as shown in Figure 1. The outer layer consisted of a higher degree of orientation with the fiber axis than turbostratic carbon layers within the fiber core [33,34,35,36]. Assisted with X-ray diffraction, Wicks and Coyle [37] found the thickness of the outer layer (*T_outer_*) for a high-strength carbon fiber was about 1 μm. Feih et al. [25] reported that the *T_outer_* of T700 carbon fiber was about 0.65 μm. In fact, *T_outer_* depends on the manufacturing process of the carbon fibers, and varies with fiber types. According to the rule of mixtures, a non-uniform stiffness distribution relative to skin–core structure owing to oxidation is shown as follows:

(1) $$\begin{array}{c} E_{av}=A_{i}E_{core}/A_{0}+\left(A_{0}-A_{i}\right)E_{surface}/A_{0}\;\;\;\\ A_{i}=\pi d_{i}{}^{2}/4,A_{0}=\pi d_{0}{}^{2}/4 \end{array}$$

where *A*_0_ and *A_i_* are the load-bearing area of the original and exposed fiber at elevated temperatures, *d*_0_ and *d_i_* are the diameter of the original and exposed fiber, *E_surface_* and *E_core_* are the Young’s modulus of the outer and core region, and *E_av_* is the average fiber modulus for the as-received fiber at room temperature. When the temperature exposure etched the carbon layer until the core region, then *d_i_* = *d_core_*, *d_core_* is the diameter of core region, and the outer layer thickness *T_outer_* = (*d*_0_ − *d_core_*)/2. It is also worth mentioning that the applicable condition of the rule of mixtures was that the modulus properties of the outer layer and core did not change during exposure. This was supported by the fact that the exposure temperatures used in this study were much lower compared to the production temperature of the carbon fiber (>1500 °C) [38,39].

### 3.2. The Modulus for Shear between Graphite Planes

**Figure 2:**
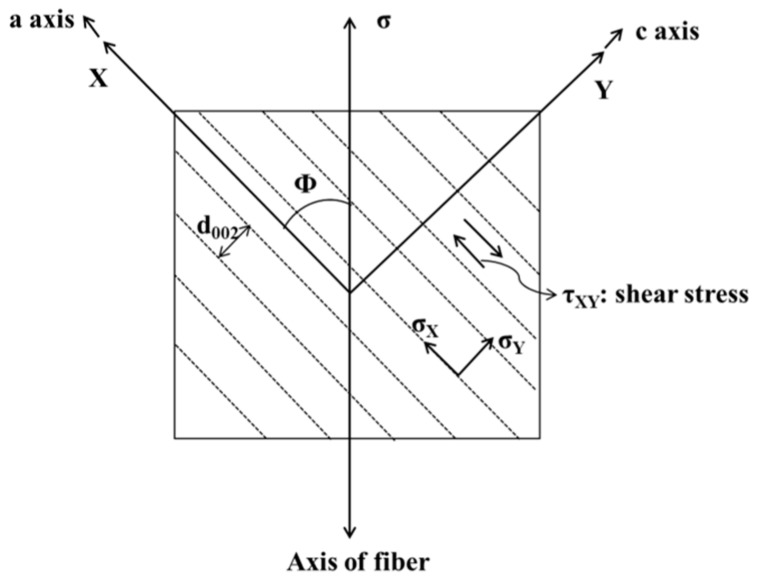
Schematic diagram including the graphitic planes at angle *ϕ* with respect to the loading direction and the stress state normal and parallel to the graphitic planes.

Figure 2 shows the graphitic planes distribution at angle *ϕ* with respect to the loading direction. The modulus for shear *G_XY_* and shear strength *τ_XY_* between graphite planes are present owing to the orientation angle *ϕ*. According to the theory of elasticity for anisotropic solids, Sauder et al. [40] deduced a theoretical model to associate the modulus for shear *G_XY_* between graphite planes with tensile modulus, as follows:

(2) $$1/E=1/E_{X}\cos^{4}\phi +1/E_{Y}\sin^{4}\phi +\cos^{2}\phi \sin^{2}\phi \left(1/G_{XY}-\nu_{\mathrm{XY}}\left(1/E_{X}+1/E_{Y}\right)\right).$$

On the basis of Equation (2), Northolt [41] proposed another model, when neglecting sin^4^ *ϕ* for well-oriented carbon fibers, presented as follows:

(3) $$1/E=1/E_{X}+\sin^{2}\phi \left(1/G_{XY}-\left(2/E_{X}+2\nu_{\mathrm{XY}}/E_{Y}\right)\right),$$

where *E* is the tensile modulus as obtained by the tensile test, *E_X_* is the Young’s modulus in the direction normal to the c-axis and is 1020 GPa, *E_Y_* is the Young’s modulus parallel to the axis and is more realistic for 20 GPa, *ν_XY_* is the Poisson ratio and is assigned as 0.25, and *G_XY_* is the modulus for shear between the graphite planes oriented normal to the c-axis. cos^2^ *ϕ*, cos^4^ *ϕ*, sin^2^ *ϕ*, and sin^4^ *ϕ* are the second and fourth moments of the orientation distribution of graphitic planes, defined as

(4) $$\cos^{n}\phi =\int_{0}^{\pi /2}I\left(\phi \right)\cos^{n}\phi \sin\phi d\phi /\int_{0}^{\pi /2}I\left(\phi \right)\sin\phi d\phi ,$$

(5) $$\sin^{n}\phi =\int_{0}^{\pi /2}I\left(\phi \right)\sin^{n}\phi \sin\phi d\phi /\int_{0}^{\pi /2}I\left(\phi \right)\sin\phi d\phi ,$$

where I(*ϕ*) is the distribution of intensity of scattering, which can be determined by wide-angle X-ray diffraction and *ϕ* is the preferred orientation angle. 

In the present paper, Northolt’s model was applied for the calculation of the shear modulus of carbon fiber. Furthermore, the shear strength *τ_XY_* is calculated by the elasticity theory for anisotropic solids, as follows:

(6) $$\tau_{XY}=\sigma \sin\phi \cos\phi ,$$

where *σ* is the tensile strength of carbon fiber per the tensile test, the orientation angle *ϕ* is 18°, and sin *ϕ* cos *ϕ* is 0.294. 

According to Equations (3) and (6), the modulus for shear *G_XY_* and shear strength *τ_XY_* between graphite layers are shown in Table 2.

## 4. Results and Discussion

### 4.1. Mechanical Properties

**Figure 3:**
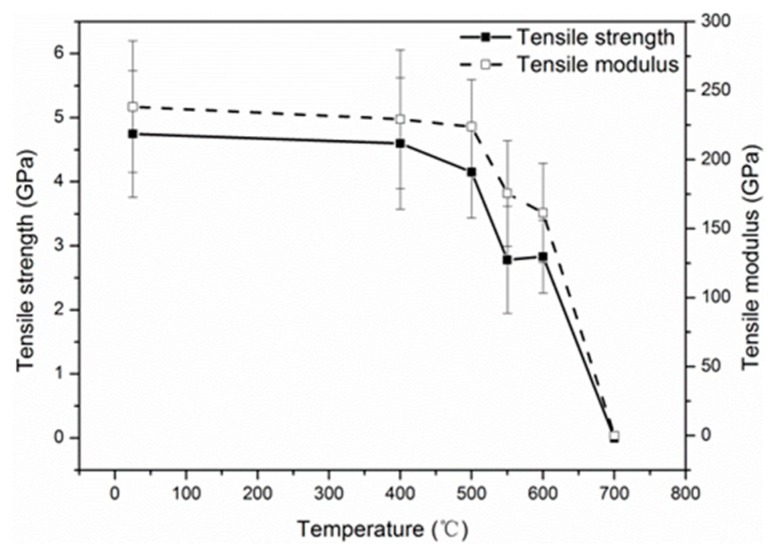
Effects of elevated temperatures on the mechanical properties of single carbon fibers. Note: the exposure time was 30 min at each temperature.

The tensile strength and modulus of single carbon fibers as a function of elevated temperatures are shown in Figure 3. As can be seen, below 400 °C, the tensile strength decreased slightly with the exposure temperatures. Then, a sharp reduction of the tensile strength was observed with the further increase of the exposure temperatures to 700 °C. For example, the carbon fiber possessed a tensile strength of about 2.78 GPa at 550 °C, with a reduction of 41.4% compared to the control fiber; at 700 °C, the carbon fibers were completely oxidized into a white-colored residue, and no strength was left at all. The same result has been reported elsewhere [26]. The tensile strength of the carbon fiber exposed at 300 °C for 10 h was still 4.58 ± 0.63 GPa, which did not bring noticeable degradation (not shown in Figure 3). In addition, the tensile modulus of carbon fiber at elevated temperatures showed a similar evolution with the tensile strength. However, the tensile modulus of the carbon fiber exposed at 300 °C for 10 h (260.6 GPa) was higher than that of control carbon fiber (238.3 GPa), and the tensile modulus increase can possibly be attributed to the removal of the surface defects by the mild oxidation [42]. The errors in the tensile properties were large, possibly because carbon fibers have nonuniform structures (e.g., skin–core structure) and the defect distribution (i.e., microcrack size and arrangement) adds data randomness and dispersion of tensile properties for carbon fibers. 

**Figure 4:**
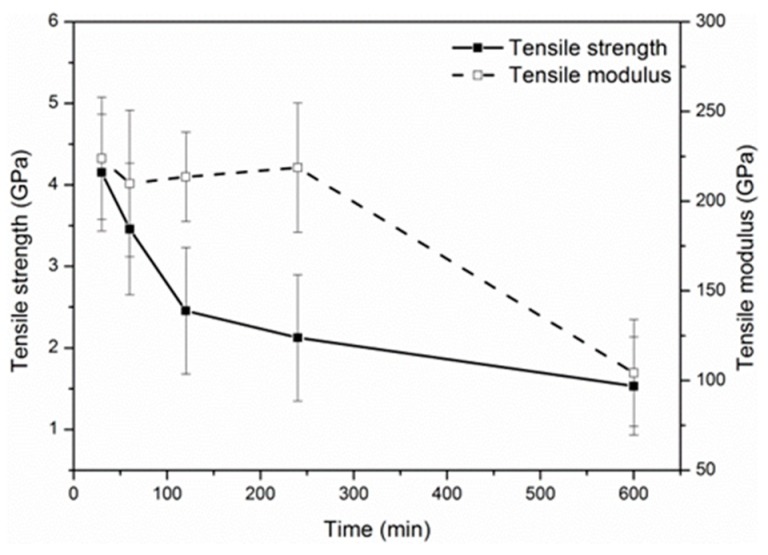
Effects of exposure time at 500 °C on the mechanical properties of single carbon fibers.

Figure 4 shows the variation of the tensile strength and modulus as a function of the exposure time at 500 °C. The exposure temperature (500 °C) was regarded as the “knee-point” temperature for the tensile degradation of carbon fibers (see Figure 3). As shown in Figure 4, the tensile strength decreased quickly in the initial exposure of two hours, and subsequently the degradation rate slowed until 10 h. The strength retention after 10 h exposure was 36.9%. On the contrary, the tensile modulus of carbon fibers remained almost unchanged in the first exposure of 4 h, but dropped by 54% after 10 h.

The tensile strength of carbon fibers before and after exposures were analyzed with the Weibull’s model based on the statistical distribution of the failure strengths, as follows [39]:

(7) $$P(\sigma )=1-\exp(-(L/L_{0})(\sigma /\sigma_{0})),$$

where *P*(*σ*) is the cumulative failure probability, *m* is the shape parameter, *σ*_0_ is the Weibull scale parameter, *L* is the carbon fiber length, and *L*_0_ is a reference length. When *L* = *L*_0_, Weibull parameters can be obtained from the conventional Weibull linear regression estimator:

(8) $$\ln(-\ln(1-P(\sigma )))=m\ln\sigma -m\ln\sigma_{0}.$$

The cumulative failure probability *P*(*σ*) at the *i*-th ranked specimen from a total of *N* specimens is obtained from the mean rank method, as follows:

(9) P(σ)=i/(N+1).

Based on Equations (8) and (9), the shape parameters (*m*) of carbon fibers before and after exposure were obtained and are shown in Table 3. It is well-known that the shape parameter *m* from Weibull’s model reflects the uniformity and reliability of the structures of a carbon fiber [43]. A greater *m* indicates a better uniformity of the carbon fiber and a smaller discrete degree of tensile strength. Meanwhile, the shape parameter *m* also reflects the distribution inhomogeneity of defects (e.g., micro-cracks) inside the materials [44].

As shown in Table 3, *m* was almost unchanged after temperature exposure for 30 min, except for the exposure at 550 °C. The greater *m* of the carbon fiber exposed at 600 °C for 30 min compared to 550 °C for 30 min was attributed to the skin–core structure of carbon fibers. Owing to oxidation at elevated temperatures, the carbon structure was etched to the core layer (600 °C; see the outer thickness in Section 4.5.1.) and the components of the core layer reacted with the oxygen to generate more ordered structures. The reaction process is called “re-oxidation” and is verified quantitatively in the modeling presented in Section 4.5.1. 

For the time exposure at 500 °C, *m* decreased continuously with increased exposure time. This was because the ordered carbon layers were etched and removed by oxidation, and more defects and inhomogeneous structures were exposed.

### 4.2. Thermal Gravimetric Analysis

**Figure 5:**
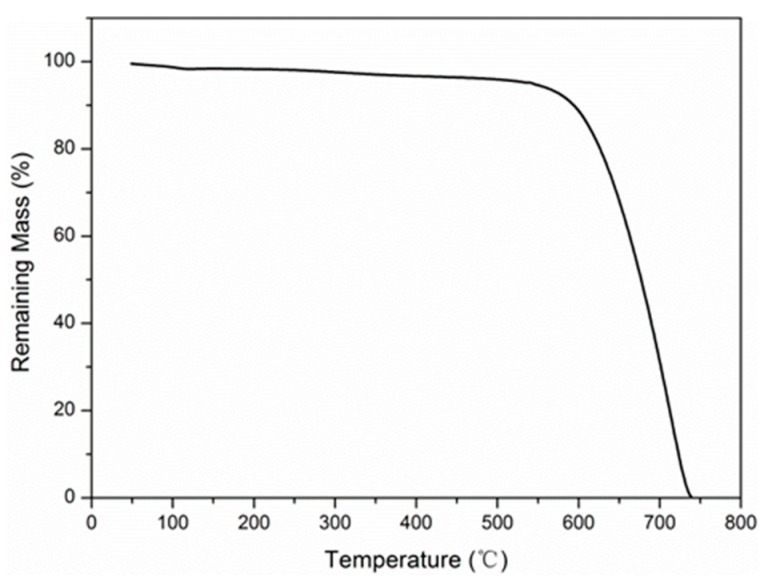
Temperature dependence of the mass evolution of carbon fiber.

**Figure 6:**
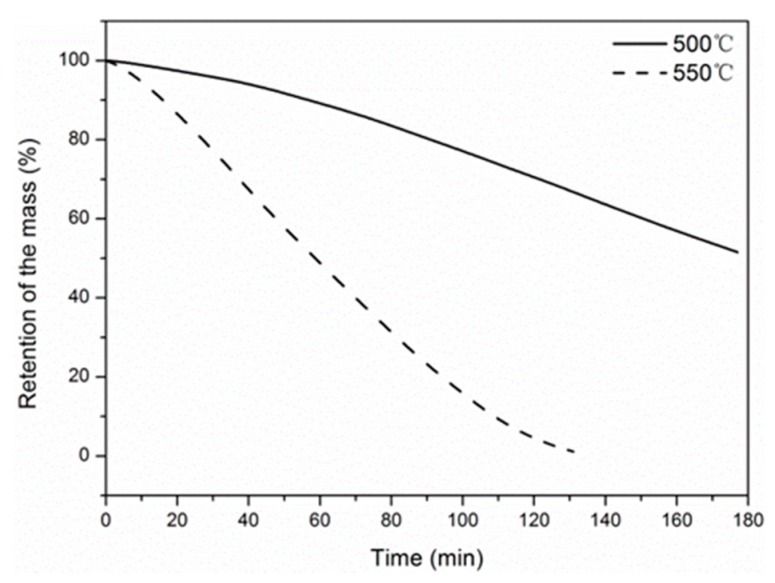
Time dependence of the mass evolution of carbon fiber at 500 and 550 °C.

The mass evolution of carbon fibers as a function of temperature in air is shown in Figure 5. As is shown, the mass of the carbon fiber decreased slightly as the temperatures increased above 500 °C, which can be attributed to the decomposition of sizing on the carbon fiber surface [25]. As the temperature reached 550 °C, significant mass loss initiated. At 739 °C, the carbon fibers were completely oxidized into carbon dioxide, which was reported elsewhere [45]. Figure 6 shows the effect of exposure time on the mass evolution of the carbon fibers at 500 and 550 °C. As is shown, the retention of the fiber mass was 51.56% after 3 h exposure at 500 °C. By comparison, complete mass loss occurred at 550 °C for 131 min. This was consistent with the variation of tensile properties at 550 °C.

### 4.3. Surface Topography

**Figure 7:**
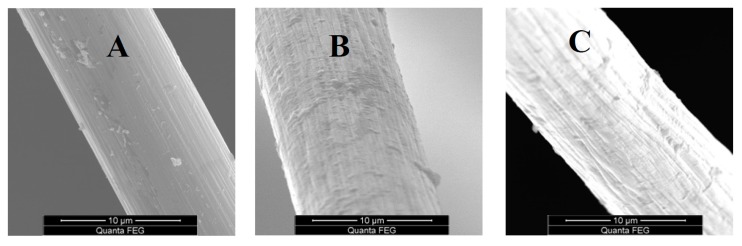
SEM pictures of carbon fibers: (**A**) original; (**B**) 500 °C for 30 min; (**C**) 500 °C for 10 h.

The surface topography of carbon fibers by SEM is shown in Figure 7. The surface of the original fibers (Figure 7A) was smooth owing to the sizing agent. With the increase of exposure temperature, the sizing agent of the carbon fiber surface started to decompose [46], leading to the appearance of shallow grooves along the fiber axis, as shown Figure 7B. The shallow grooves aligned along the fiber axis decreased when the exposure temperature and time was enough, for example 600 °C–30 min or 500 °C–10 h (Figure 7C). This was attributed to the core layer carbon structures exposed at 600 °C–30 min or 500 °C–10 h being re-oxidized, generating more ordered carbon structures. For the carbon fiber, oxidation occurred from skin to core layer with the increase of exposure temperature and time, leading to different fiber diameters and structures, including skin, skin–core interface, and core layer.

**Figure 8:**
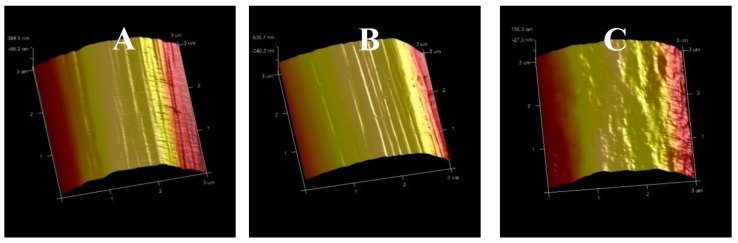
AFM pictures of carbon fibers: (**A**) original; (**B**) 500 °C for 30 min, and (**C**) 550 °C for 30 min.

Figure 8 shows the surface topography of carbon fibers by AFM. It is clear that there were narrow grooves (Figure 8A,B) in the carbon fiber surface [47], as shown in the SEM photos. With the exposure temperature increasing to 550 °C, the surface grooves of carbon fiber disappeared (Figure 8C). A similar phenomenon was also presented at 600 °C for 30 min and 500 °C for 10 h.

### 4.4. Elements and Functional Groups

Surface element contents and functional groups of original and exposed carbon fibers are shown in Table 4 and Table 5. The content of carbon element decreased obviously with the increase of exposure temperature and time. The oxygen content and the oxygen/carbon ratio increased, even more than one exposed at 500 °C for 10 h. As shown in Table 5, the percentage of C–C skeleton decreased greatly with the exposure temperatures and times, and the contents of C–OH (C–O–C) and C=O increased. In addition, the content of COOH had a negligible change except in the exposure at 500 °C for 10 h.

**Figure 9:**
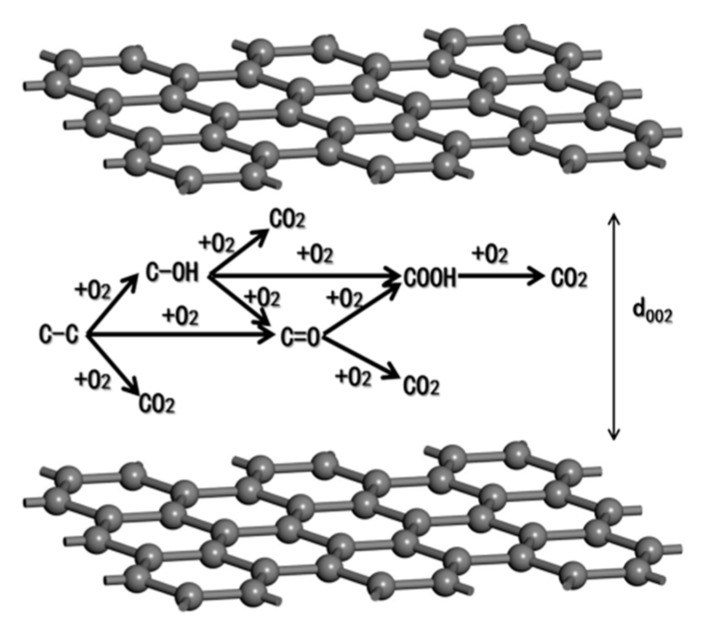
Transformation schematic diagram of oxygen-containing functional groups between the graphite layers.

When the carbon fibers were exposed to elevated temperatures, a large amount of carbon skeletons were destroyed due to oxidation, which led to a decrease of carbon content and an increase of oxygen. After experiencing enough exposure time (e.g., 300 °C for 10 h), the surface sizing agent of carbon fibers was fully removed, so the nitrogen decreased sharply. Figure 9 shows the transformation schematic diagram of oxygen-containing functional groups between the graphite layers. At elevated temperatures, the C–C skeleton fractured owing to oxidation, and the resulting carbon atoms combined with the oxygen into other containing oxygen groups (C–OH, C–O–C, and C=O). Furthermore, these oxygen-containing groups reacted with oxygen again to generate COOH or carbon dioxide. When the generation rate and reaction rate were basically at equilibrium, the percentage of COOH remained basically unchanged.

**Figure 10:**
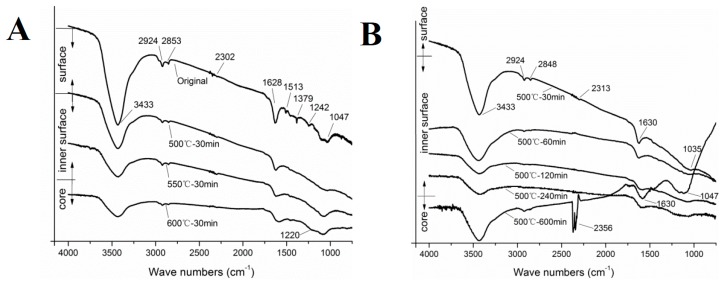
FTIR spectra of carbon fibers: (**A**) exposed to different temperatures for 30 min; (**B**) exposed at 500 °C for different times.

FTIR spectra of carbon fibers at different exposure temperatures and times are shown in Figure 10. The original spectra had several obvious absorption peaks: 2924 cm^−1^, 2853 cm^−1^, 1628 cm^−1^, 1513 cm^−1^, 1379 cm^−1^, 1242 cm^−1^, and 1047 cm^−1^, where 2924 cm^−1^, and 2853 cm^−1^ are the stretching vibration double peaks of C–H; 1628 cm^−1^ is the C=C skeleton stretching vibration peak; 1513 cm^−1^ is C–N=O stretching vibration peak; 1379 cm^−1^ is O–H deformation stretching vibration peak; 1242 cm^−1^ is C=O stretching vibration peak; and 1047 cm^−1^ is the C–O stretching vibration peak. 

By comparing the original carbon fiber with others exposed to different temperature–time treatments (Figure 10A), the stretching vibrations located at 1513 cm^−1^ corresponding to C–N=O vibration were the vibrations of sizing agent composition of the carbon fiber surface. With increased exposure temperatures, the sizing agent decomposed and the vibration disappeared. It was observed that the C=C skeleton stretching vibration (1628 cm^−1^) and the C–H vibration (2924 cm^−1^, 2853 cm^−1^) were gradually weakened, which had a good agreement with the quantitative analysis results from XPS. It is worth mentioning that the carbon fiber exposed at 600 °C for 30 min led to a new weak C=O stretching vibration at 1220 cm^−1^. This vibration was formed by the core layer structure exposed to oxygen and the occurrence of the “re-oxidation”. Figure 10B shows the FTIR spectra of carbon fibers exposed at 500 °C. The obvious new N–H vibration located at 2356 cm^−1^ (500 °C for 10 h) was originally a part of the composition of carbon fiber precursor [48]. Furthermore, the inadequate “pre-oxidation” led to the core layer structure of carbon fibers inherited partly from the precursor’s structure. This also verified that the surface structure of carbon fiber exposed at 500 °C for 10 h was a part of the core layer.

### 4.5. Modeling of Tensile Properties

#### 4.5.1. Tensile Modulus

##### Exposure Temperatures

Different carbon layer structures were revealed with the increase of exposure temperature. The structure of carbon fibers could be divided into skin layer and core layer based on the outer layer thickness *T_outer_*. For each carbon layer, an exponential decay model was put forward to describe the radial distribution of tensile modulus, as follows:

(10) $$E(d^{2})\;=E_{surface}-A\exp(Bd^{2})\;(A,B=\mathrm{constant}),$$

where *E*(*d*^2^) and *d* are the tensile modulus and diameter of each layer, *E_surface_* is the surface layer modulus based on the assumption of a completely homogeneous structure. The expression *A*exp(*Bd*^2^) is put forth as an attenuation form of the tensile modulus from the outer layer to the core layer. If *A* = 0, the tensile modulus of carbon fiber remains unchanged with the increase of exposure temperature, which means the skin–core structure disappears owing to the complete pre-oxidation during the carbon fiber production process. 

By fitting, we obtain that:

(11) $$E(d^{2})\;=334.11-333.76\exp(-0.026d^{2}).$$

**Figure 11:**
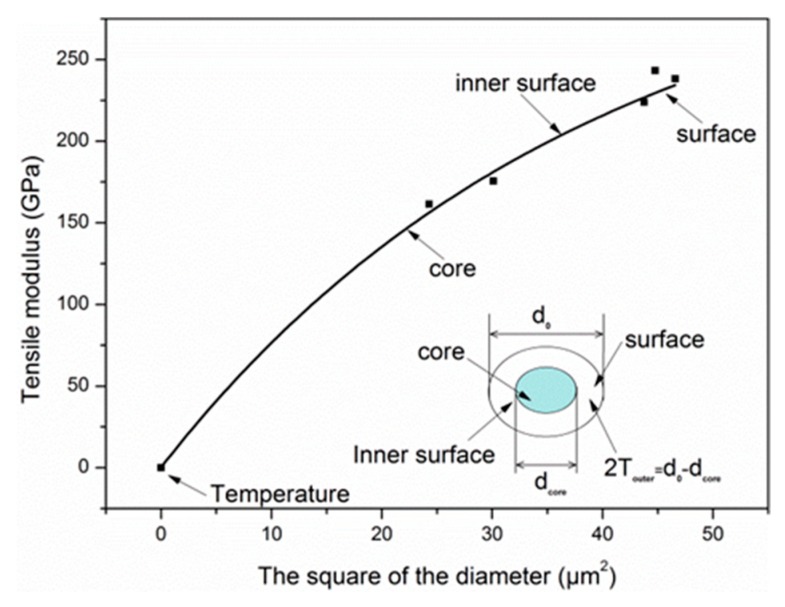
The dependence of the exposure temperature on the radial distribution of tensile modulus of carbon fibers.

The obtained *E_surface_* was 334.11 GPa. By comparison, the *E_surface_* was calculated as 315 GPa for T700 carbon fiber by Feih et al. [25]. The fitting curve is shown in Figure 11, and has high R-squared value (R^2^ = 0.99).

When oxidation etched the carbon layer until the core region, the tensile modulus of the core layer was calculated according to Equation (11) as follows:

(12) $$E(d^{2}{}_{core})\;=334.11-333.76\exp(-0.026d^{2}{}_{core}),$$

where *E*(*d_core_*^2^) and *d_core_* are the tensile modulus and diameter of the core layer, respectively. 

Substituting Equations (11) and (12) into Equation (1), the outer layer thickness *T_outer_* of carbon fibers was obtained by using Matlab software (MathWorks, Natick, MA, USA), as follows:

(13) $$d_{core}=5.190\;\mu m,\;T_{outer}=0.818\;\mu m$$

In contrast, Wicks and Coyle [37] measured the *T_outer_* of a high-strength carbon fiber to be about 1 μm using X-ray diffraction, and the value of *T_outer_* was 0.65 μm for T700 carbon fiber in the study of Feih et al. [25]. From the high R-squared value and the comparison of our work with that of others, the exponential models can describe the degradation of tensile modulus of carbon fibers at elevated temperatures.

With the increase of exposure temperature, the diameter of carbon fiber was etched by oxidation from the outer layer to the core layer. Similarly, an exponential decay model was applied to describe the variation of diameter, as follows:

(14) $$d^{2}(T)=d_{n}{}^{2}-A\exp(BT)\;(A,B=\mathrm{Constant}),$$

where *d_n_* is the nominal diameter of carbon fiber as received (7.00 μm), with some deviations with the *d*_0_ allowed, and *T* is the exposure temperature from 25 to 700 °C.

By fitting, the variation of diameter with the exposure temperature was determined:

(15) $$d^{2}(T)=49.00-0.2217\exp(0.0077T).$$

**Figure 12:**
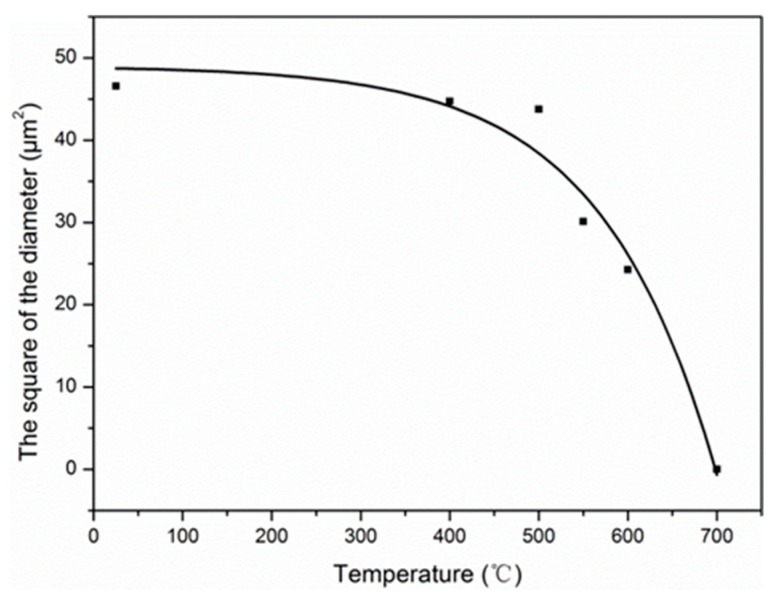
The effect of exposure temperature on the square of the diameter of carbon fibers.

As shown in Figure 12, the good degree of fitting (R^2^ = 0.96) indicates that the exponential decay model can do well in reflecting the decrease of diameter with the exposure temperature. 

Through the superposition of Equations (11) and (15), the tensile modulus degradation model with exposure temperature is presented as follows:

(16) E=334.11−100.17exp(−0.216+0.0198exp(0.00611T)).

**Figure 13:**
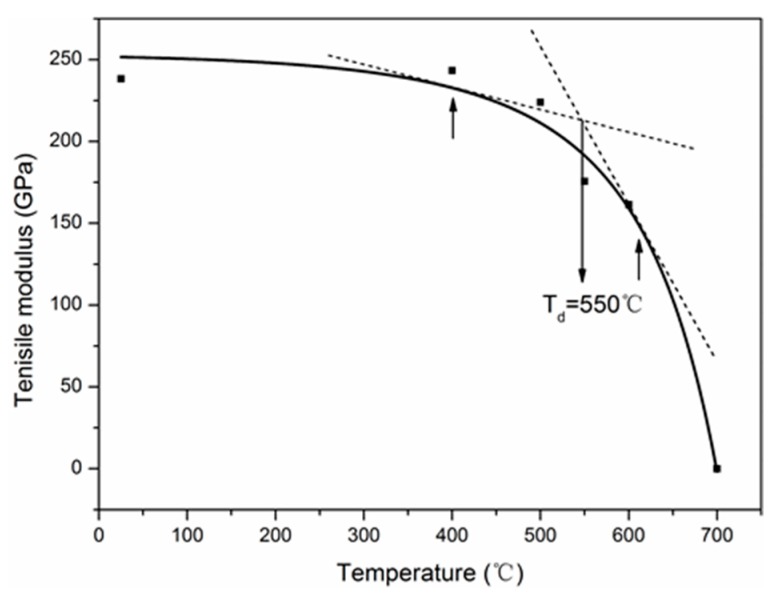
Variation of tensile modulus with the exposure temperature.

The degradation curve of tensile modulus with exposure temperature is shown in Figure 13. Let *E* = 0, *T* = *T_u_* = 699.4 °C, where *T_u_* is the ultimate exposure temperature, which was verified by the presence of residual “white-colored ashes” found when fibers were exposed to 700 °C for 30 min in the laboratory. Besides, it can be seen that the obvious degradation of tensile modulus started from 400 to 600 °C. Within this temperature range, the tangent intersection of degradation curve (Figure 13) was close to 550 °C, which was defined as the decomposition temperature of carbon fiber denoted by *T_d_*.

Here, the normalized oxidation degree (*OD_T_*) related to the exposure temperature is introduced to quantitatively analyze the effect of oxidation, as follows:

(17) $$OD_{T}=\frac{E_{av}-E}{E_{av}}\times 100\%.$$

**Figure 14:**
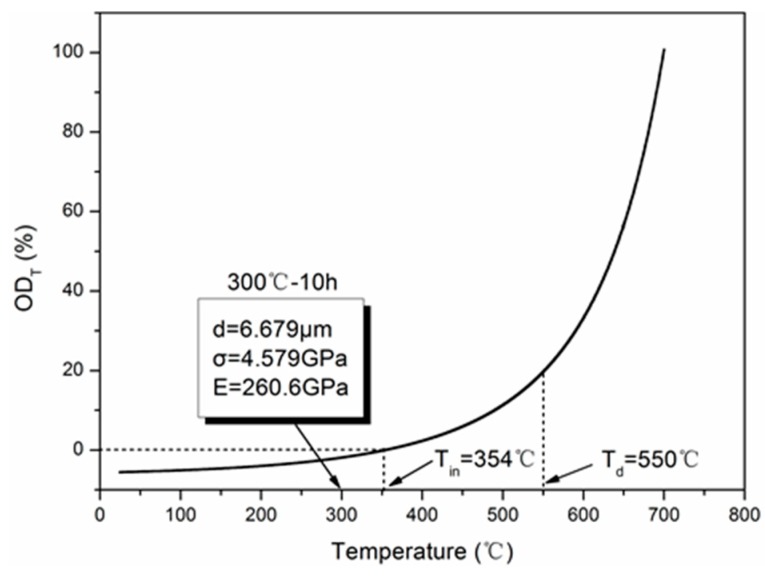
Variation of oxidation degree (*OD_T_*) versus the exposure temperature.

Figure 14 shows the normalized oxidation degree curve. It can be seen that oxidation actually initiated at 354 °C and decomposed at 550 °C. When the exposure temperature is under the initial oxidation temperature, it can be predicted that no degradation occurs, which was verified by the result that no obvious degradation of tensile modulus was observed for the exposure at 300 °C for 10 h. When the exposure temperature exceeded the decomposition temperature of carbon fiber, obvious degradation of tensile modulus appeared based on the tensile test results.

##### Exposure Time

According to the equivalence of exposure temperature and time on the tensile modulus, the degradation model of tensile modulus exposed at 500 °C for different times was obtained when omitting the deduction process, as follows:

(18) $$E(t)=0.00125\exp(14.037+8.899\times 10^{-5}t-3.440\times 10^{-7}t^{2})-1349.66.$$

**Figure 15:**
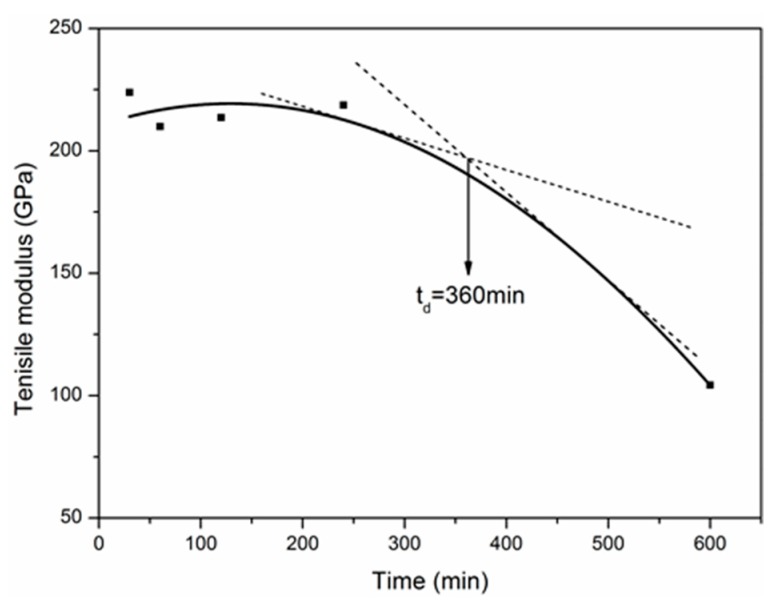
Variation of tensile modulus with the exposure time.

**Figure 16:**
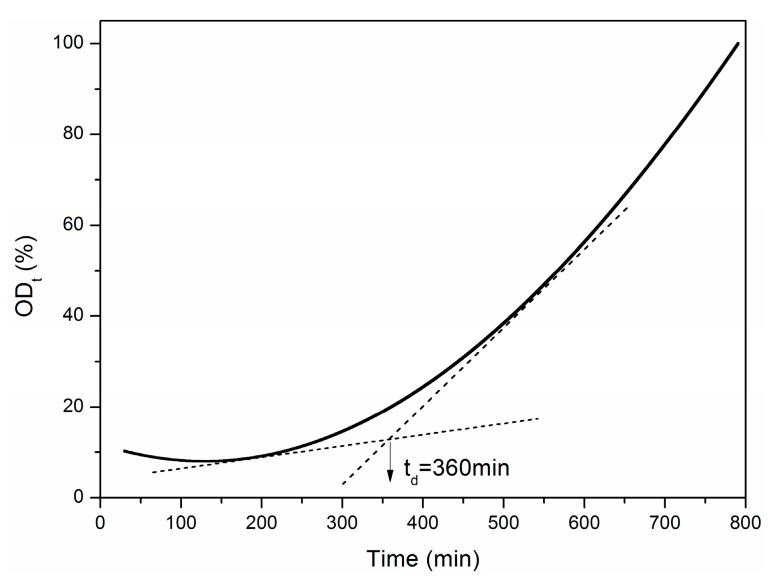
Variation of oxidation degree (*OD_t_*) versus the exposure time.

The degradation curve of tensile modulus with exposure time is shown in Figure 15. Let *E* = 0, *t* = *t_u_* = 790.92 min = 13.18 h. *t_u_* is the ultimate exposure time at 500 °C. Similarly, the decomposition time *t_d_* was obtained to be about 6 h (Figure 15). At the same time, the normalized oxidation degree (*OD_t_*) related to the exposure time is shown in Figure 16.

In summary, *T_d_* and *t_d_* could be used to characterize the response of the carbon fibers to fire at a specific exposure temperature and time. When the *T_d_* is higher and the *t_d_* is longer, the fire endurance of carbon fiber is better.

##### Time–Temperature Equivalence

**Figure 17:**
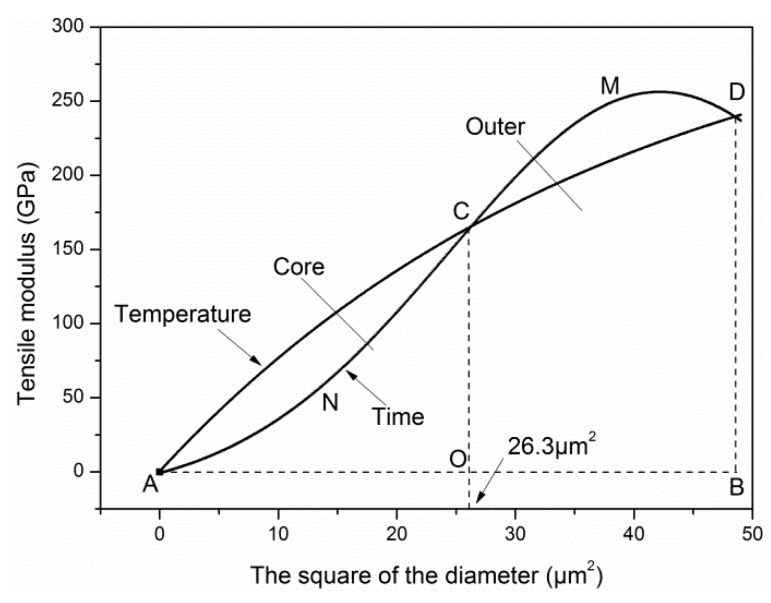
Time–temperature equivalence on the radial distribution of tensile modulus.

After obtaining the degradation model of tensile modulus exposed for different temperatures and times, the time–temperature equivalence of tensile modulus on cross-section distribution was studied. Figure 17 shows the variation curves of tensile modulus with the square of the diameter exposed to different temperatures and times. Obviously, the two curves had three intersection points, which were A (*d*^2^ = 0.0 μm^2^), C (*d*^2^ = 26.3 μm^2^), and D (*d*^2^ = 48.5 μm^2^), respectively. Furthermore, the effect of exposure temperature and time on tensile modulus could be characterized through the areas *A_ACDB-T_* and *A_ANCMDB-t_* surrounded by the two curves and two straight lines (*E* = 0 GPa and *d*^2^ = 48.5 μm^2^). Based on these areas, the time–temperature equivalence can be discussed quantitatively, as follows:

(19) $$A_{ACDB-T}=7125.1,A_{ANCMDB-t}=6953.9,\frac{A_{ACDB-T}-A_{ANCMDB-t}}{A_{ACDB-T}}=0.024\approx 0.$$

From almost identical areas surrounding by the two curves with the *d*^2^ axis, the equivalence of exposure temperature and time on tensile modulus was verified. It is worth mentioning that point C was a dividing point, and the corresponding diameter was marked as *d_dp_* (5.128 μm). It indicated that when *d* < *d_dp_*, exposure temperature was the major influencing factor of tensile modulus, and when *d* > *d_dp_*, exposure time was the major influencing factor. Besides, it can be observed that *d_dp_* = 5.128 μm ≈ *d_core_* = 5.190 μm, which meant the C point was also the cut-off point of the skin layer and core layer of carbon fibers. 

**Figure 18:**
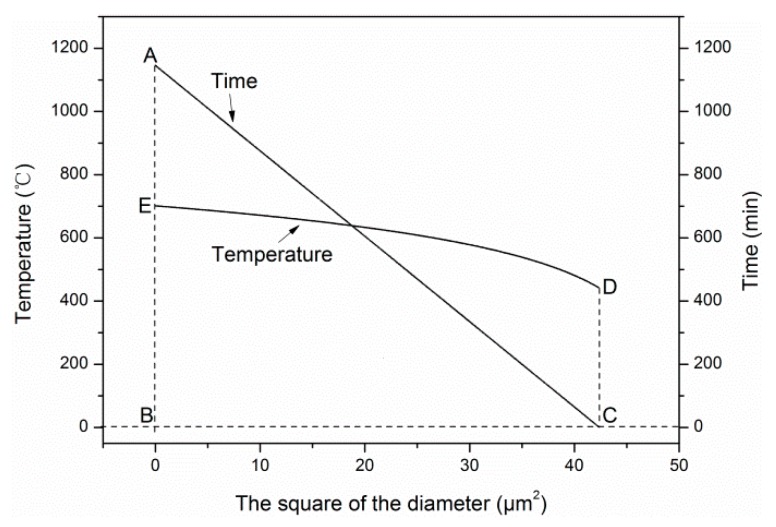
Equivalence of exposure temperature and time on the diameter.

Similarly, the equivalence of exposure temperature and time on the diameter was analyzed quantitatively, and is shown in Figure 18.

(20) $$A_{ABC-t}=24279.1,A_{EBCD-T}=25893.1,\frac{A_{EBCD-T}-A_{ABC-t}}{A_{EBCD-T}}=0.062\approx 0$$

**Figure 19:**
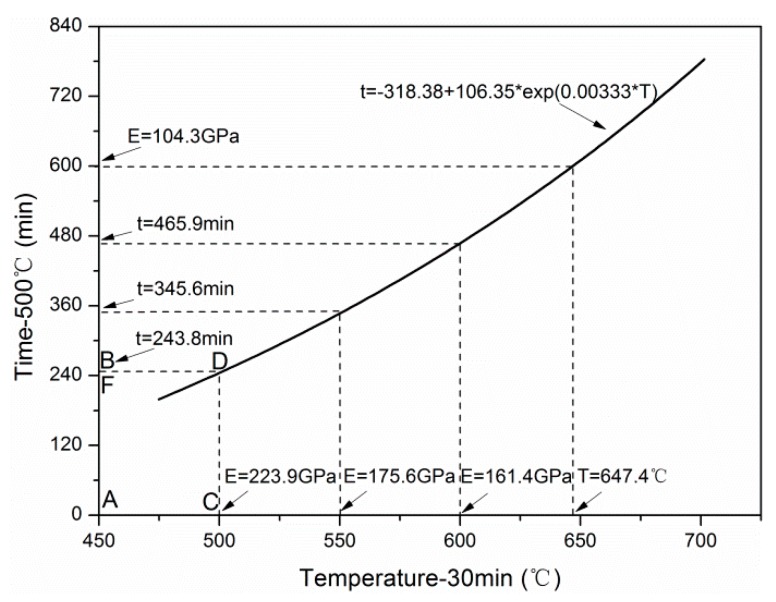
Time–temperature equivalence equation.

After the discussion on the equivalence of exposure temperature and time, the time–temperature equivalence equation of tensile modulus degradation was deduced by eliminating the intermediate variables *OD_T_* (Figure 14) and *OD_t_* (Figure 16), and is shown in Figure 19. A good equivalence was observed for the variation of tensile modulus. Note that the C point of 500 °C (30 min) on the abscissa corresponded to the B point of 500 °C (243.8 min) on the ordinate, which could be explained by the fact that the modulus located in the area surrounded by ABDC kept almost unchanged. For example, the *E*_C_ (500 °C–30 min) was 223.9 GPa, close to the 218.7 GPa of the *E*_F_ (500 °C–240 min).

##### Arbitrary Exposure Temperatures and Times

When the carbon fibers are exposed to fire, the uncertainty of exposure temperature and time provides the impetus to obtain a degradation model for the tensile modulus under the interaction of arbitrary exposure temperature and time. Based on the above analysis, the normalization method was adopted to obtain the *OD*_(*T*,*t*)_ at any temperature and time. Furthermore, the *OD*_(*T*,*t*)_ was applied to characterize the degradation of tensile modulus.

Firstly, *OD_T_* was normalized to acquire the temperature normalizing factor *T_nf_* by normalizing the data point of 500 °C (30 min), and the other exposure temperature points took it as a reference. Similarly, the time normalizing factor *t_nf_* was obtained by normalizing the data point of 30 min (500 °C) and the other exposure time points took it as a reference. Then, the mixture rule was used to obtain the final *OD*_(*T*,*t*)_ as follows:

(21) $$OD(T,t)=A\times OD_{T}t_{nf}+(1-A)\times OD_{t}T_{nf}$$

where *A* is the influence coefficient. Known by the time–temperature equivalence, let *A* = 0.5. *OD*_(*T*,*t*)_ was finally obtained. It can be seen that the higher *T* and longer *t* resulted in greater *OD*_(*T*,*t*)_. For example, *OD*_(*T*,*t*)_ was basically in flat state before 500 °C. The transition period between 500 °C and 600 °C brought in a slow increase of *OD*_(*T*,*t*)_. When the temperature was more than 600 °C, *OD*_(*T*,*t*)_ increased sharply until a limit temperature was reached (700 °C) and the tensile modulus was completely lost.

#### 4.5.2. Tensile Strength

##### Exposure Temperatures

Griffith micro-crack theory states that the interior cracks of brittle materials extend in an unstable way when the elastic strain energy released by crack extension overcomes the material’s resistance. With the spread of crack extension, the initial stress concentration forms and intensifies until the complete fracture in the lower nominal stress. In terms of carbon fiber material, fiber or loading direction has an angle *ϕ* with the load-carrying graphitic planes (Figure 2), which introduces shear stress between the graphite layers. When the shear strength between the graphitic planes of carbon fiber is lower than the shear stress, cracks expand rapidly until the formation of a through-wall crack. 

In the present paper, the oxidation of carbon fibers at elevated temperatures led to more interior cracks and drastically weakened the shear strength *τ_XY_* (material resistance). When allowing for the relatively low exposure temperature compared to the temperature in the production process (*T* > 1500 °C) of the carbon fibers, the shear strain *γ* between the graphite layers may be almost constant, which indicated the shear strength *τ_XY_* was proportional to *G_XY_*, as follows:

(22) $$\tau_{XY}=0.197G_{XY}-4.898.$$

A high R-squared (R^2^ = 0.93) verified the linear relationship between *τ_XY_* and *G_XY_*. According to the previous study, the dependence of *G_XY_* on *E* is shown as follows:

(23) $$G_{XY}=0.0333E+23.474.$$

Based on Equations (6), (14), (20) and (21), the degradation of tensile strength model (*σ*–*T*) was obtained as follows:

(24) σ=6.517−2.228exp(−0.216+0.0198exp(0.00611T)).

**Figure 20:**
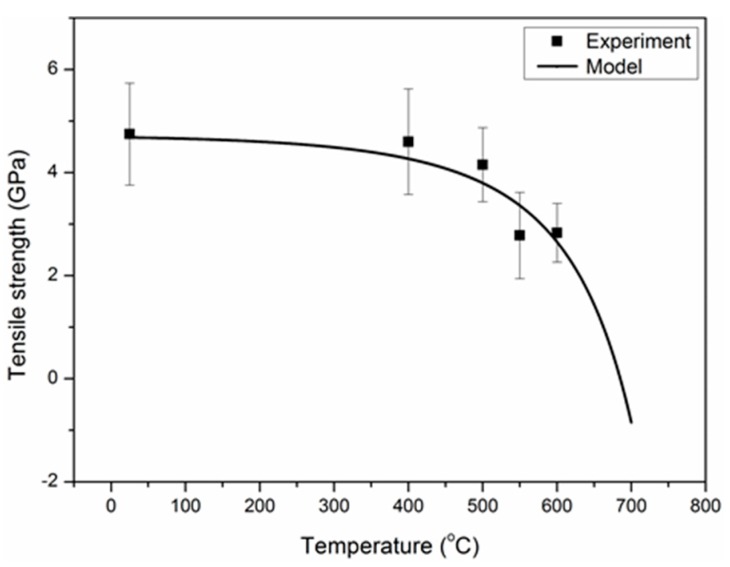
The dependence of the exposure temperature on the tensile strength.

The degradation curve of tensile strength exposed at elevated temperatures is shown in Figure 20. As shown, the model was in good agreement with the experimental data when considering the allowable experimental error.

##### Exposure Time

**Figure 21:**
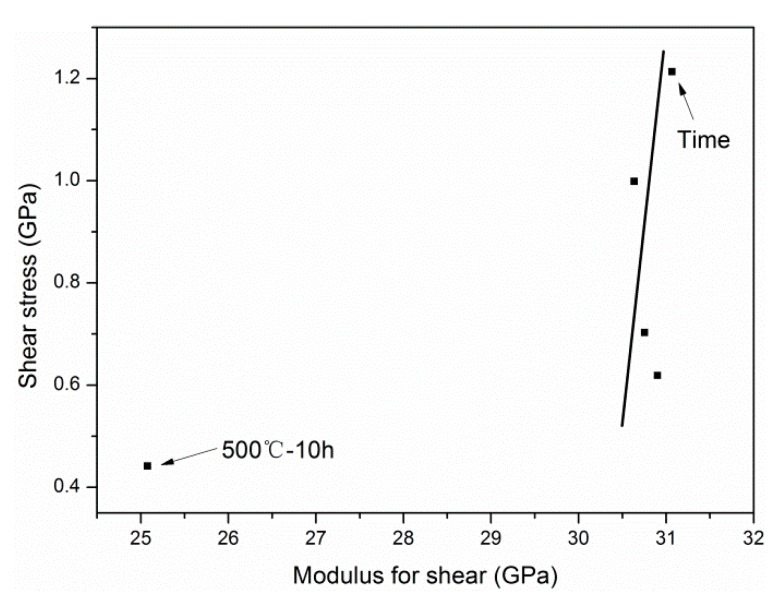
The dependence of the exposure time on the relationship between the modulus for shear and the shear strength between the graphite layers.

A similar methodology was also applied for obtaining the degradation model of tensile strength for different exposure times. However, the linear dependence was not applicable to describe the relationship between *τ_XY_* and *G_XY_* when exposed at 500 °C for 10 h, as shown in Figure 21. The constant shear strain condition did not apply to this point. The possible reason for this was that the internal micro-cracks of the carbon layer exposed at 500 °C for 10 h became larger and expanded rapidly to the critical crack size during tensile tests, leading to the lower shear strain. Based on the relationship among the *E*, *G_XY_*, and *τ_XY_*, an approximate model was put forward to characterize the degradation process, as follows:

(25) $$\tau_{XY}=A\exp(B+Ct+Dt^{2})+F,A,B,C,D,F=\mathrm{Constant}.$$

**Figure 22:**
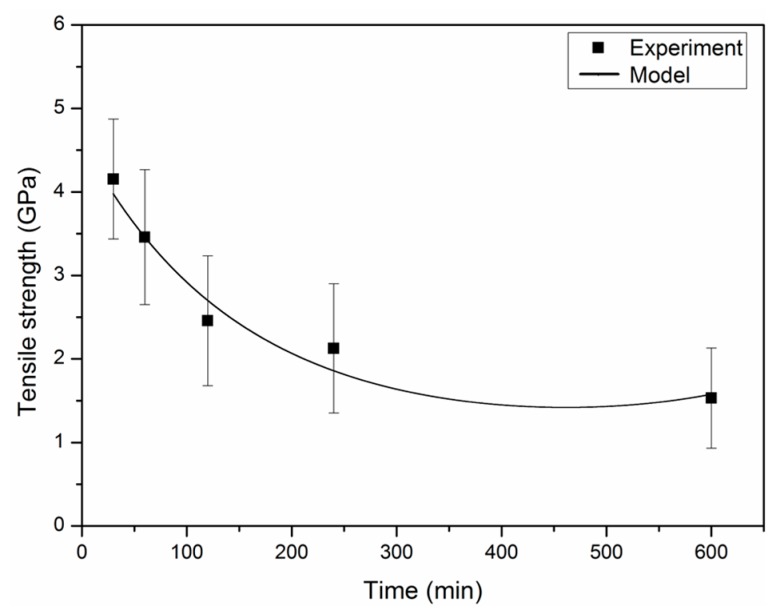
The dependence of the exposure time on the tensile strength.

By fitting, *A* = 0.987, *B* = 0.310, *C* = −0.0052, *D* = 5.6336 × 10^−6^, *F* = 0.0118. For *σ*–*t*, *A* = 3.357, *B* = 0.310, *C* = −0.0052, *D* = 5.6336 × 10^−6^, *F* = 0.040. Accordingly, the obtained *σ*–*t* is displayed in Figure 22.

## 5. Conclusions

In the present paper, the degradation behavior of carbon fiber tensile properties at elevated temperatures was studied and modeled. The mass loss, surface morphology, and elements and functional groups of carbon fibers were characterized. The rule of mixture was applied to deduce the tensile modulus degradation model. The elastic mechanics theory was cited to obtain the degradation model of tensile strength. The following conclusions can be drawn based on the experimental and analysis results.

The tensile strength and modulus of the carbon fibers decreased remarkably when the exposure temperature exceeded 500 °C. Oxidation at elevated temperatures etched the carbon layer from the skin to core of a carbon fiber, leading to mass loss. The skin–core structure of carbon fiber was revealed, and the thickness of the outer layer was derived to be 0.818 μm. From the tensile modulus degradation model, the ultimate exposure temperature of carbon fiber was predicted to be 699.4 °C for 30 min and the ultimate exposure time was 13.2 h at 500 °C. Furthermore, the time–temperature equivalence equation of tensile modulus was proposed. The normalization method was adopted to obtain the tensile modulus variation at any exposure temperature and time. The linear dependence was established for the shear strength and shear modulus between the graphite layers. A degradation model of tensile strength exposed to elevated temperature was put forward, and the proposed model had good agreement with the experimental results. 

In this paper, the degradation rule of tensile properties of PAN-based carbon fibers at elevated temperatures was obtained. The degradation mechanism of carbon fibers at elevated temperatures was revealed through the analysis of mass loss, surface morphology, and elements and functional groups. The rule of mixtures was adopted to quantitatively characterize the skin–core structures of carbon fibers. An exponential decay model was put forward to describe the degradation behavior of tensile modulus exposed to different temperatures and times. From the elastic mechanics theory for anisotropic solids, the degradation model of tensile strength exposed to elevated temperature was confirmed. The authors expect the research methodology in this paper will be applied for the degradation process of tensile properties for other carbon fibers with skin–core structure.

## Figures and Tables

**Table 1:** Exposure conditions of carbon fiber-reinforced polymers (CFRPs) at elevated temperatures for engineering applications.

Composite Type	Temperature Range (°C)	Maximum Exposure Time (h)	Fire Protection Coating
CFRP bars [27]	0–400	2	Inorganic mortar cladding
CFRP strips [28]	0–1000	3	Intumescent coating
CFRP strips [29]	0–1100	5	Fire protective boards
CFRP laminates [6]	0–1000	2.5	Vermiculite-perlite mortar fire protection
CFRP laminates [30]	0–165	8	-
CFRP laminates [31]	0–600	1	Laminate plaster board/intumescent paint

**Table 2:** Shear properties between graphite layers calculated by Equations (3) and (6).

Samples	*G_XY_* (GPa)	*τ_XY_* (GPa)	Samples	*G_XY_* (GPa)	*τ_XY_* (GPa)
Original ^a^	31.584	1.399	500 °C–30 min	31.067	1.213
400 °C–30 min	31.452	1.346	500 °C–1 h ^b^	30.635	0.999
550 °C–30 min	29.363	0.803	500 °C–2 h	30.755	0.703
600 °C–30 min	28.729	0.833	500 °C–4 h	30.901	0.619
700 °C–30 min	0	0	500 °C–10 h	25.076	0.442

^a^ The original is the untreated sample (control sample); ^b^ 500 °C–1 h: 500 °C is the exposure temperature, 1 h is the exposure time.

**Table 3:** Shape parameter (*m*) of carbon fibers.

Samples	Shape Parameter (*m*)	Samples	Shape Parameter (*m*)
Original	5.217	500 °C–1 h	5.229
400 °C–30 min	5.560	500 °C–2 h	3.684
500 °C–30 min	5.483	500 °C–4 h	3.388
550 °C–30 min	3.816	500 °C–10 h	2.570
600 °C–30 min	5.293	-	-

**Table 4:** Surface element contents of original and exposed carbon fibers determined by XPS.

Samples	C (%)	O (%)	N (%)	O/C (%)
Original	69.8	25.61	4.59	36.69
300 °C–10 h	75.45	23.77	0.79	31.50
500 °C–30 min	61.95	34.05	4.00	54.96
550 °C–30 min	58.86	35.26	5.89	59.90
500 °C–2 h	61.05	34.33	4.62	56.23
500 °C–10 h	34.15	61.26	4.59	179.36

**Table 5:** Surface functional groups of control and exposed carbon fibers determined by XPS.

Samples	C–C		C–OH (C–O–C)		C=O		COOH (R)	
	Binding Energy (eV)	Percentage (%)	Binding Energy (eV)	Percentage (%)	Binding Energy (eV)	Percentage (%)	Binding Energy (eV)	Percentage (%)
Original	284.13	72.12	285.64	22.57	287.52	5.31	-	-
300 °C–10 h	284.60	76.51	286.11	14.21	288.45	9.28	-	-
500 °C–30 min	284.54	71.44	286.11	17.65	287.76	5.56	289.72	5.35
550 °C–30 min	284.41	51.53	285.72	34.82	287.47	10.24	288.92	3.41
500 °C–2 h	284.22	41.83	285.20	40.41	287.10	12.11	289.19	5.65
500 °C–10 h	284.26	37.61	285.36	47.59	287.35	13.97	289.71	0.83
